# *Campylobacter coli* infection in pet birds in southern Italy

**DOI:** 10.1186/s13028-016-0271-y

**Published:** 2017-01-06

**Authors:** Ludovico Dipineto, Luca Borrelli, Antonino Pace, Violante Romano, Stefano D’Orazio, Lorena Varriale, Tamara Pasqualina Russo, Alessandro Fioretti

**Affiliations:** Department of Veterinary Medicine and Animal Productions, Università di Napoli Federico II, Via della Veterinaria 1, 80137 Naples, Italy

**Keywords:** *Campylobacter* spp., *Campylobacter coli*, Avian species, Pet birds, Zoonosis

## Abstract

Avian species are considered as the main reservoir of *Campylobacter* spp. However, few data are available on the presence of this microorganism in pet birds. This study was therefore performed to determine the prevalence of *Campylobacter* spp. in pet birds bred in southern Italy. Faecal samples were collected from 88 cages housing different species of pet birds and examined by bacteriological culture and polymerase chain reaction. A total of 13.6% of the cage samples were positive for *Campylobacter coli*. Other *Campylobacter* spp. were not found. The study shows that *C. coli* can be isolated from the cages of apparently healthy pet birds, which should therefore be considered as potential carriers of *C. coli* and a possible source of infection for humans and companion animals.

## Findings

Thermotolerant *Campylobacter* spp., mainly *Campylobacter jejuni* and *Campylobacter coli*, are the most commonly reported bacteria in enteric infections in humans. The incidence of human campylobacteriosis has increased in both developed and developing countries over the last 10 years [1]. These bacterial species colonize the intestinal mucosa of most warm-blooded animals, including food-producing animal species and humans [2].

Several avian species are considered the main reservoirs of *Campylobacter* spp. [3, 4]. Nevertheless, current scientific knowledge on the presence of *Campylobacter* spp. in pet birds is scarce. To address this lack of information, the present study was undertaken to assess the presence of *Campylobacter* spp. in pet birds bred in southern Italy.

The study was carried out from July to December 2015 in 14 privately owned bird farms located in the Campania region, southern Italy. Sampling was conducted with the approval of the owners. In each farm, the bird population ranged from 20 to 100 birds. Pooled faecal samples were obtained from the floor of 88 cages with birds belonging to the families of Estrildidae (33 cages with 118 birds), Fringillidae (28 cages with 64 birds) and Psittacidae (27 cages with 43 birds) (Table 1). The cage was used as an epidemiological unit, and each cage housed from one to five birds. All birds were apparently in healthy condition and none received any antimicrobial treatment during the study period.

**Table 1:** Family and species of birds examined, related bird populations and number of cages tested with percentage of cages being positive for *Campylobacter coli*

Family	Birds tested	Bird population/number of cages tested	Number of positive/pooled fecal samples tested (%)
Estrildidae	*Erythrura gouldiae*	67/22	0/22 (0%)
	*Lonchura striata domestica*	16/4	0/4 (0%)
	*Taeniopygia guttata*	35/7	7/7 (100%)
Fringillidae	*Carduelis carduelis*	18/9	0/9 (0%)
	*Serinus canaria*	46/19	0/19 (0%)
Psittacidae	*Agapornis* spp.	20/10	2/10 (20%)
	*Amazona* spp.	6/6	3/6 (50%)
	*Arinae* subfamily	6/3	0/3 (0%)
	*Cacatuidae* family	2/2	0/2 (0%)
	*Loriinae* subfamily	2/1	0/1 (0%)
	*Melopsittacus undulatus*	2/1	0/1 (0%)
	*Nymphicus hollandicus*	2/1	0/1 (0%)
	*Psittacus erithacus*	3/3	0/3 (0%)
Total		225/88	12/88 (13.6%)

Bird population refers to the total number of birds housed in the total number of cages examined

Before the collection of faecal samples, a sheet of sterile aluminum foil was placed under the grid of each cage overnight. Faecal samples were then collected by sterile cotton tipped swabs. Each sample swab was stored in Amies Charcoal Transport Medium (Oxoid, Basingstoke, UK) at 4 °C, transported to the laboratory, and analyzed within 2 h of collection. Samples were inoculated into Bolton selective enrichment broth (Oxoid) and incubated at 42 °C for 48 h under microaerobic conditions provided by CampyGen (Oxoid). Subsequently, each sample was streaked onto *Campylobacter* blood-free selective agar (modified charcoal cefoperazone deoxycholate agar; Oxoid) with the corresponding supplement (SE 155; Oxoid). The plates were examined for typical *Campylobacter* colonies after additional incubation at 42 °C for 48 h under microaerobic conditions. The suspected colonies were purified on sheep blood agar (Oxoid) and finally incubated for 24 h at 42 °C. Colonies comprising curved or spiral motile rods were examined by phase contrast microscopy, presumptively identified as *Campylobacter* spp. and submitted to a multiplex polymerase chain reaction (PCR) analysis following the procedures described by Gargiulo et al. [5].

All positive isolates were tested for the antimicrobial susceptibility by using the disk diffusion method and breakpoints as suggested by Sifré et al. [6]. Because few breakpoints are available for *Campylobacter* spp., only ciprofloxacin (5 μg), erythromycin (15 μg), and tetracycline (30 μg) were tested.

Twelve out of the 88 cages [13.6%; 95% confidence interval (CI) 7.6–23.0%] were positive for a *Campylobacter* spp., which in all cases was identified as *C. coli*. Seven out of 33 cages with bids of the Estrildidae family (21.2%; 95% CI 9.6–39.4%) and five out of 27 cages with birds of the Psittacidae family (18.5%; 95% CI 7.0–38.8%) were infected with *C. coli*, while all cages (n = 28) with birds of the Fringillidae family were negative (Table 1). Out of the 14 farms, five farms had infected birds (35.7%; 95% CI 14.0–64.4%). All *C. coli* isolates were sensitive to erythromycin and resistant to tetracycline and ciprofloxacin.

Except for a study reporting a prevalence of campylobacteriosis in pet birds in Argentina to 19.0% [7], data on the occurrence of campylobacteriosis in pet birds are scarce. In our study, 13.6% of the cage samples were found positive for *C. coli*. The majority of the positive samples (7/12) originated from *Taeniopygia guttata* species in which *C. coli* was found in all samples (7/7 species samples) followed by *Amazona* spp. (3/12 positive samples; 3/6 species samples) and *Agapornis* spp. (2/12 positive samples; 2/10 species samples).

This study shows that *C. coli* may be excreted in the faeces of apparently healthy pet birds. Pet birds may be a potential source of *C. coli* transmission to humans and the risk of transmission of antimicrobial resistant bacteria between pet birds and other animal species and humans should be considered. The adoption of good hygiene practices when handling pet birds should be promoted.
